# Supplementary material for: Evolution of a biological thermocouple by adaptation of cytochrome c oxidase in a subterrestrial metazoan, Halicephalobus mephisto
Source: Commun Biol. 2024 Sep 28;7:1214. doi: 10.1038/s42003-024-06886-z (PMC11439043; doi:10.1038/s42003-024-06886-z)
Supplement: Supplementary file 2 — Supplementary Information [file 42003_2024_6886_MOESM2_ESM.pdf]

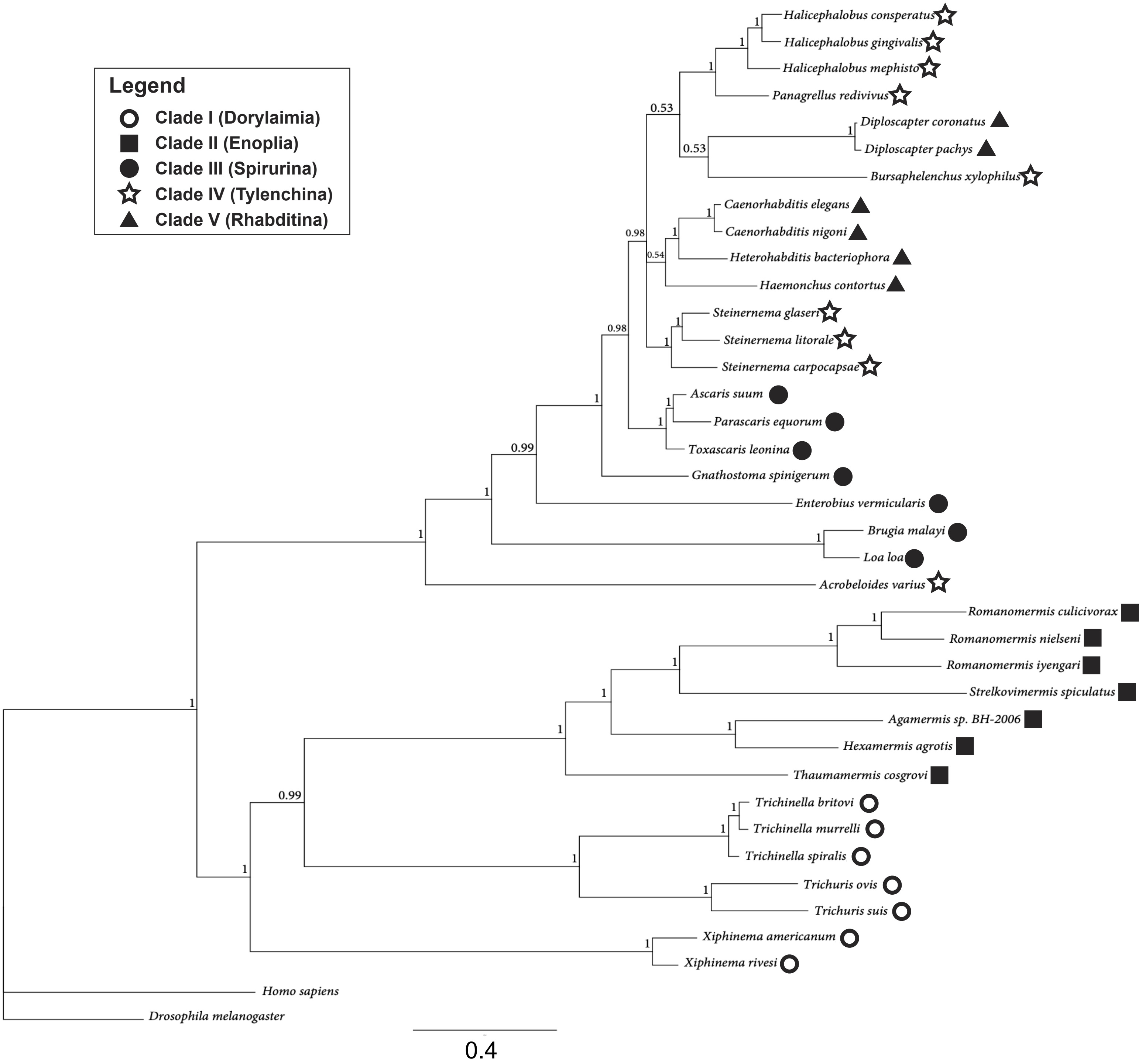

Figure S1. Bayesian catenated mitochondrial protein tree. Numbers on branches indicate posterior probabilities. Scale bar indicates substitutions per site.

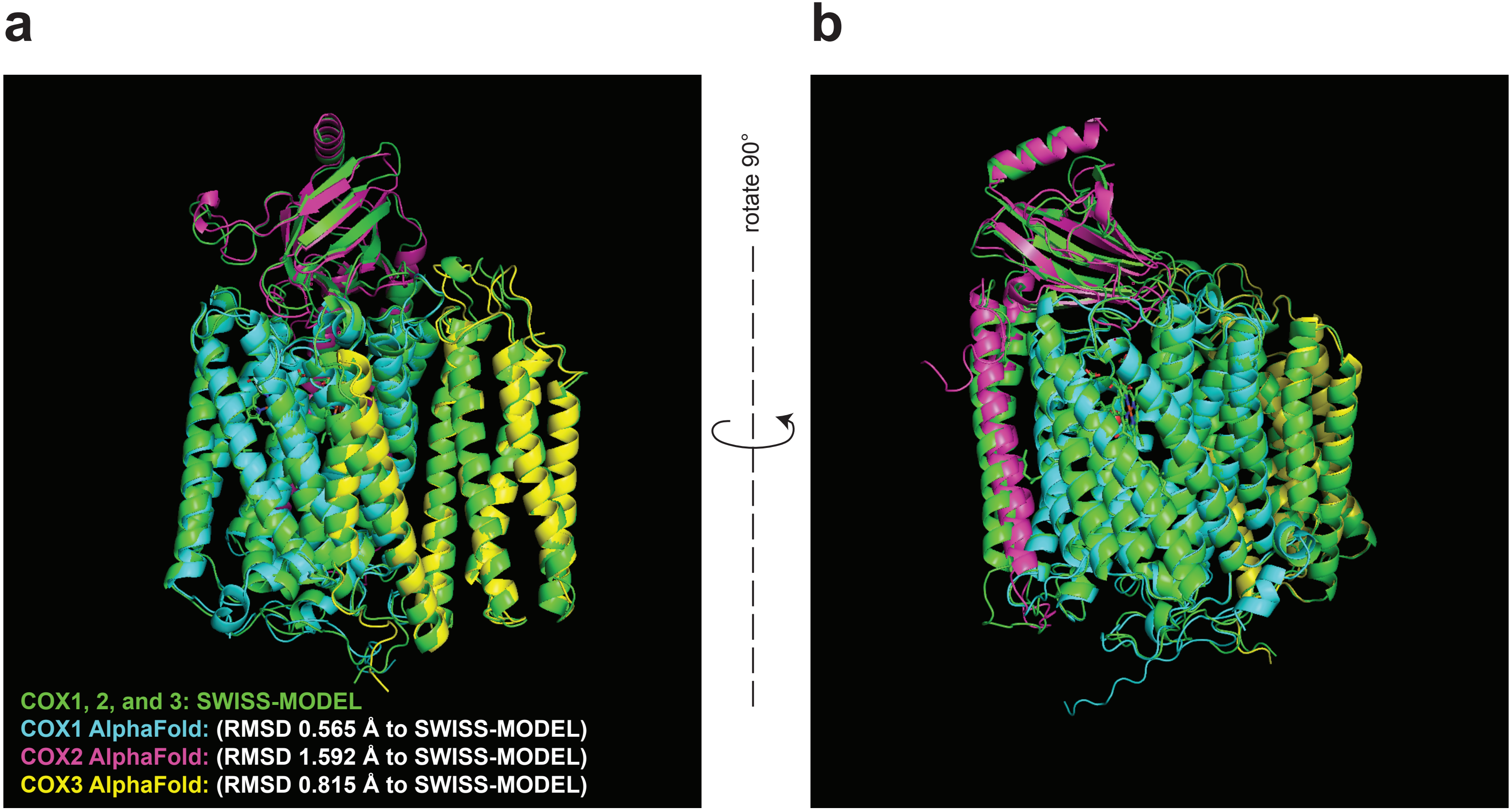

Figure S2.Superimposition of SWISS-MODEL homology model of *H. mephisto* COX1, COX2, and COX3 with AlphaFold predictions. The SWISS-MODEL structure is green, while the AlphaFold COX1 structure is cyan, the AlphaFold COX2 structure is purple, and the AlphaFold COX3 structure is yellow. Both panels (a) and (b) show the same structural alignment but rotated 90°.

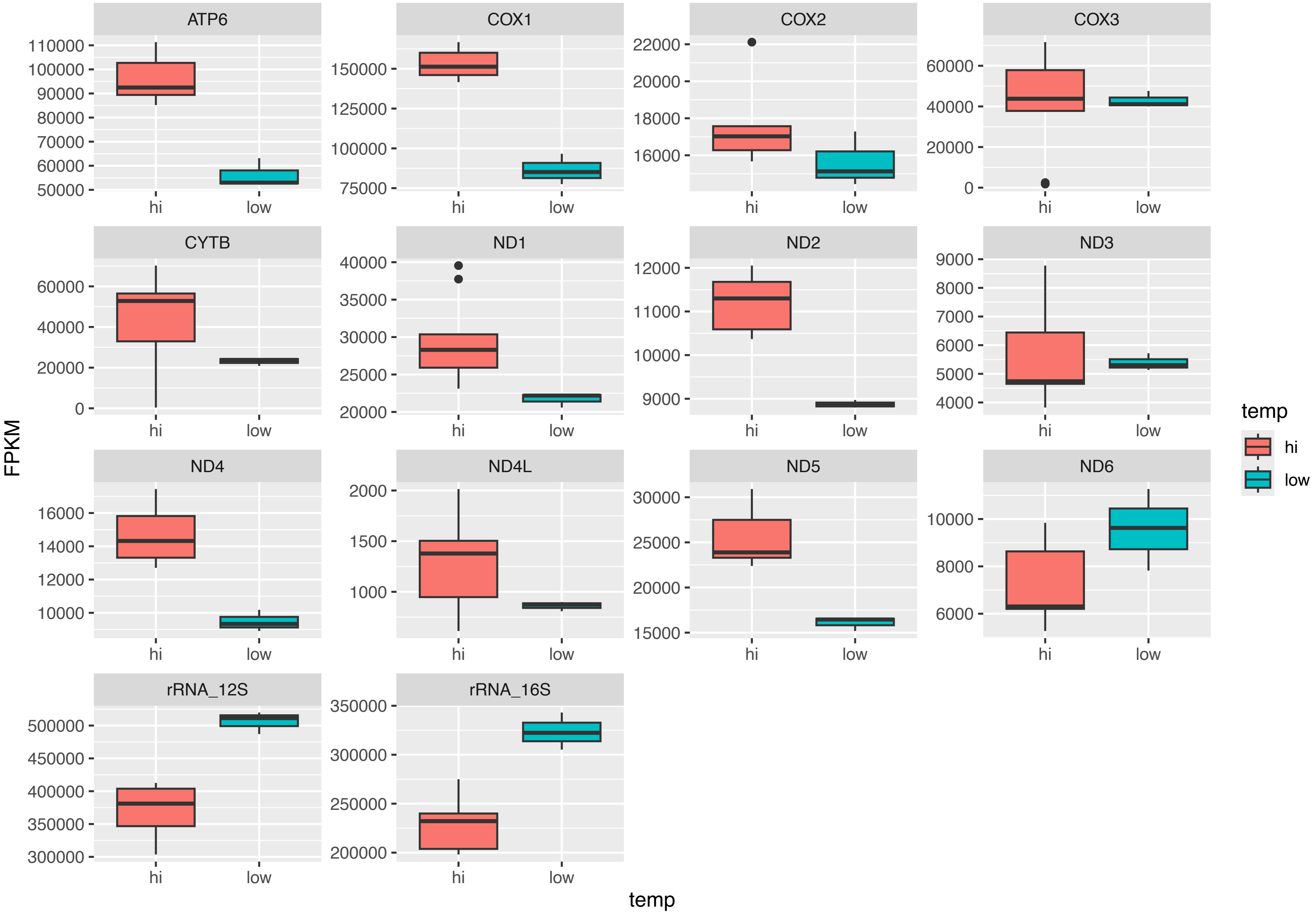

Figure S3. RNAseq expression of mitochondrial protein-coding genes at 25°C (low) and 38-40°C (high). All temperature-driven changes of expression are less than 2-fold. Shown is average FPKM, Fragments per Kb per Million mapped reads. Sequencing was performed for n=3 experimental replicates for low temperature and n=6 experimental replicates for high temperature.

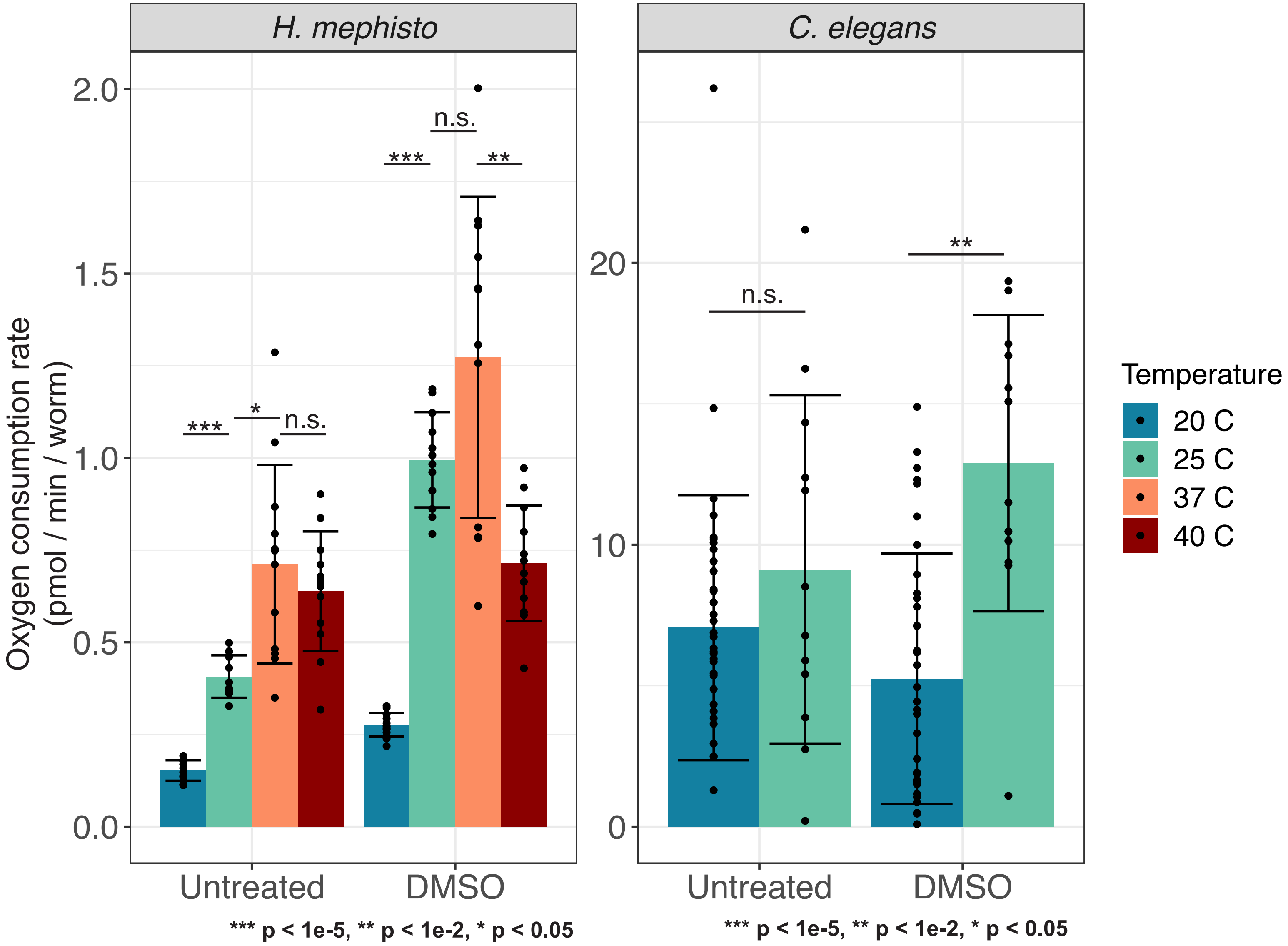

Figure S4. DMSO effect on oxygen consumption rate of *H. mephisto* and *C. elegans*. Shown is the mean with error bars representing standard deviation. All p-values calculated by pairwise comparisons using Wilcoxon rank sum exact test.

Table S1. Accession numbers used in this study.

Species	Clade	Accession Number	Genome Length
Trichinella britovi	I	NC_025750	16,421
Trichinella murrelli	I	NC_025751	16,592
Trichinella spiralis	I	NC_002681.1	16,706
Trichuris ovis	I	NC_018597	13946
Trichuris suis	I	NC_017747.1	14,436
Xiphinema americanum	I	NC_005928	12,626
Xiphinema rivesi	I	NC_033869	12,624
Agamermis sp. BH-2006	II	NC_008231	16,561
Hexamermis agrotis	II	NC_008828	24,606
Romanomermis culicivorax	II	NC_008640	26,194
Romanomermis iyengari	II	NC_008693	18,919
Romanomermis nielseni	II	NC_008692	15,546
Strelkovimermis spiculatus	II	NC_008047	18,030
Thaumamermis cosgrovei	II	NC_008046	20,013
Ascaris suum	III	NC_001327	14,248
Brugia malayi	III	AF538716	13,657
Enterobius vermicularis	III	NC_056632.1	14,010
Gnathostoma spinigerum	III	NC_027726	14,079
Loa loa	III	HQ186250	13,590
Parascaris equorum	III	NC_036427	13,899
Toxascaris leonina	III	NC_023504	14,310
Bursaphelenchus xylophilus	IV	AP017463	15,197
Halicephalobus consperatus	IV	MN207311.1	13,886
Halicephalobus mephisto	IV	N/A	14,349
Halicephalobus gingivalis	IV	KM192363	13,375
Panagrellus redivivus	IV	AP017464	13,908
Acrobeloides varius	IV	MK559448	17650
Steinernema carpocapsae	IV	AP017465.1	13,924

Steinernema glaseri	IV	AP017466.1	15,182
Steinernema litorale	IV	AP017468.1	21,403
Caenorhabditis elegans	V	NC_001328	13,794
Caenorhabditis nigoni	V	KP259621	13,856
Diploscapter coronatus	V	LC213018	13,378
Diploscapter pachys	V	N/A	13,393
Haemonchus contortus	V	NC_010383	14,055
Heterorhabditis bacteriophora	V	NC_008534	18,128
Drosophila melanogaster	Ecdysozoa	NC_024511	19,524
Homo sapiens	Vertebrate	NC_012920	16,569
