# Supplementary material for: Evolution of a biological thermocouple by adaptation of cytochrome c oxidase in a subterrestrial metazoan, Halicephalobus mephisto
Source: Commun Biol. 2024 Sep 28;7:1214. doi: 10.1038/s42003-024-06886-z (PMC11439043; doi:10.1038/s42003-024-06886-z)
Supplement: Supplementary file 3 — Description of Additional Supplementary Files [file 42003_2024_6886_MOESM3_ESM.pdf]

Description of Additional Supplementary Files

File name: Supplementary Data 1

Description: Source data for all graphs in the manuscript.
